# Age is not a primary risk factor for anterior cruciate ligament injury—A comprehensive review of anterior cruciate ligament injury and reinjury risk factors confounded by young patient age

**DOI:** 10.1002/ksa.12646

**Published:** 2025-03-18

**Authors:** Bálint Zsidai, Ramana Piussi, Philipp W. Winkler, Armin Runer, Pedro Diniz, Riccardo Cristiani, Eric Hamrin Senorski, Volker Musahl, Michael T. Hirschmann, Romain Seil, Kristian Samuelsson

**Affiliations:** ^1^ Sahlgrenska Sports Medicine Center Gothenburg Sweden; ^2^ Department of Orthopaedics, Institute of Clinical Sciences, Sahlgrenska Academy University of Gothenburg Gothenburg Sweden; ^3^ Department of Orthopedics Skåne University Hospital Malmö/Lund Sweden; ^4^ Unit of Physiotherapy, Department of Health and Rehabilitation Institute of Neuroscience and Physiology, Sahlgrenska Academy University of Gothenburg Gothenburg Sweden; ^5^ Department for Orthopaedics and Traumatology, Kepler University Hospital GmbH Johannes Kepler University Linz Linz Austria; ^6^ Department of Sports Orthopaedics, Klinikum rechts der Isar Technical University of Munich Munich Germany; ^7^ Department of Orthopaedic Surgery Centre Hospitalier de Luxembourg – Clinique d'Eich Luxembourg Luxembourg; ^8^ Luxembourg Institute of Research in Orthopaedics, Sports Medicine and Science (LIROMS) Luxembourg Luxembourg; ^9^ Luxembourg Institute of Health (LIH) Luxembourg Luxembourg; ^10^ Department of Bioengineering and iBB – Institute for Bioengineering and Biosciences, Instituto Superior Técnico Universidade de Lisboa Lisbon Portugal; ^11^ Department of Molecular Medicine and Surgery, Section of Sports Medicine Karolinska Institutet Stockholm Sweden; ^12^ Stockholm Sports Trauma Research Center (SSTRC), FIFA Medical Centre of Excellence Stockholm Sweden; ^13^ Department of Orthopaedic Surgery UPMC Freddie Fu Sports Medicine Center University of Pittsburgh Pittsburgh Pennsylvania USA; ^14^ Department of Orthopedic Surgery and Traumatology, Head Knee Surgery and DKF Head of Research Kantonsspital Baselland Bruderholz Switzerland; ^15^ Department of Orthopaedics Sahlgrenska University Hospital Mölndal Sweden

**Keywords:** ACL‐R failure, anatomical, bone morphology, physiological, revision surgery, washout

## Abstract

Revision surgery after anterior cruciate ligament reconstruction (ACL‐R) is hypothesized to be the result of an interplay between factors associated with the anatomy, physiological characteristics and environment of the patient. The multifactorial nature of revision ACL‐R risk is difficult to quantify, and evidence regarding the independent roles of potentially important variables is inconsistent throughout the literature. Young patient age is often cited as one of the most prominent risk factors for reinjury after ACL‐R. However, the association between a non‐modifiable variable such as patient age and revision ACL‐R risk is likely to be a spurious correlation due to the confounding effect of more important variables. From the perspective of healthcare professionals aiming to mitigate revision ACL‐R risk through targeted interventions, awareness of factors like generalized joint hypermobility, bone morphology, muscle strength imbalances, and genetic factors is critical for the individualized risk assessment of patients with ACL injury. The aim of this current concepts article is to raise awareness of the essential anatomical, physiological, and activity‐related risk factors associated with ACL injury and reinjury risk that are likely captured and confounded by patient age.

**Level of Evidence:**

Level V.

**Abbreviations**
ACL: anterior cruciate ligament
ACL‐R: anterior cruciate ligament reconstruction
CT: computed tomography
GJH: generalized joint hypermobility
LFC: lateral femoral condyle
LFCR: Lateral femoral condyle ratio
LTAD: lateral tibiofemoral articular distance
MMP: matrix metalloproteinase
MRI: magnetic resonance imaging
NW: notch width
NWI: notch width index
PTS: posterior tibial slope
RFD: rate of force development
RTS: return to sport
VEGFA: vascular endothelial growth factor A

## INTRODUCTION

The prevention of anterior cruciate ligament (ACL) reinjury after ACL reconstruction (ACL‐R) requires a comprehensive understanding of the interplay between several modifiable and non‐modifiable risk factors in a heterogeneous patient population [58, 93, 94]. Over the past two decades, an increasing number of studies performed based on data queried from large scale, prospectively collected registry data have pinpointed young patient age as one of the major predictors of ACL revision, while patients 20 years or older display a marked reduction in ACL revision risk [58, 119, 132]. While decreasing activity level with age may theoretically reduce the exposure of patients to ACL revision risk, we believe that the sharp decrease in revision risk in patients older than 20 years is more likely due to the elimination of patients with an inherent risk for repeat ACL injuries—due to overlooked anatomic, physiologic and genetic factors—from the studied patient populations [61, 92, 110]. Patients at high risk for ACL revision tend to suffer primary ACL injuries early during their athletic careers [118, 119, 132], followed shortly by second and third repeat ACL injuries [86], which may ultimately force these individuals to retire from competition‐level sports. Consequently, patients predisposed to ACL reinjury due to modifiable and non‐modifiable factors are likely to undergo multiple revision ACL‐Rs at a young age. However, it is essential to differentiate the selective attrition of young patients due to underlying biological risk factors from the spurious correlation of ACL reinjury with patient age. The presented current concepts article reviews the essential anatomic, physiologic and genetic risk factors for ACL injury and reinjury that are likely captured and overshadowed by patient age and encourages risk assessment of young patients with ACL injury based on the discussed factors.

## GENERALIZED JOINT HYPERMOBILITY (GJH) AND KNEE HYPEREXTENSION

GJH refers to a clinical phenotype associated with hyperextensibility in multiple synovial joints, due primarily to genetic factors affecting the structural integrity of collagen [14, 85]. The clinical assessment of patients with GJH is performed with a series of joint mobility tests, which are used to calculate the Beighton score (Table 1) based on the number of positive tests recorded [7, 154]. Depending on the patient's age, a Beighton score of 4 or 5 out of 9 is used as a threshold to clinically confirm the GJH phenotype, with the occasional use of an injury allowance point by some authors in the event of known previous injury to the contralateral knee [19, 154]. Previous studies have reported the role of GJH as an independent risk factor for the incidence of primary ACL injury [126] and may also be associated with increased rotatory knee laxity in the setting of primary ACL injury [125]. Another registry study reported an inferior 2‐year return to sport (RTS) rate (49.2% vs. 57.3%) and inferior knee extension strength in patients with GJH compared with patients without GJH following primary ACL‐R [79]. Further studies corroborate the impact of GJH on second ACL injury, graft failure and suboptimal postoperative subjective knee function after primary ACL‐R [72, 155]. When considering the prevalence of ACL graft retear, excessive graft laxity and contralateral ACL tear, one study found a compounded ACL‐R failure rate of 34.1% at a mean 6‐year follow‐up in patients with ACL‐R [72]. Additionally, another recent study found a 5.53‐fold increased odds of a second (ipsilateral or contralateral) ACL injury in patients with GJH compared to patients without GJH within 12 months of RTS after primary ACL‐R [155]. Additionally, the same study determined a 4.24‐fold lifetime hazard ratio of second ACL injury after RTS [155]. While further research is required to optimize surgical management and postoperative rehabilitation protocols for patients with GJH and ACL injury, integration of the Beighton score to screen patients at risk for ACL reinjury is recommended [154].

**Table 1:** Clinical tests for the assessment of generalized joint hypermobility based on the Beighton score [154].

Greater than 90° of passive dorsiflexion and hyperextensibility of metacarpophalangeal joint V	1 point right side/1 point left side
Ability to passively oppose thumb against forearm flexors	1 point right side/1 point left side
Greater than 10° of passive elbow hyperextension	1 point right side/1 point left side
Greater than 10° of passive knee hyperextension	1 point right side/1 point left side
Ability to actively flex trunk with palms flat against the ground.	1 point
Beighton score	Sum of positive tests (out of 9 points)

Physiologic knee hyperextension is defined as an asymptomatic and painless recurvatum of the knee joint beyond the knee range of motion. Physiologic knee hyperextension is considered an independent risk factor for ACL injury [97], with a potential contribution to inferior postoperative subjective and objective patient outcomes following RTS after ACL‐R [66, 69, 72]. While a clear threshold for the magnitude of knee hyperextension associated with an increased risk of ACL reinjury has been elusive, one recent study found that contralateral knee hyperextension of 6.5° and beyond was correlated with 14.6‐fold odds of hamstring tendon ACL graft retear [44]. Additionally, hyperextension of the contralateral knee is associated with greater magnitudes of anterior tibial translation of the ACL‐injured knee after ACL‐R [127], and knee hyperextension was determined a risk factor for postoperative ACL graft laxity [152]. Patients with hyperextensible knees may therefore be susceptible to a greater risk of graft failure and residual anteroposterior knee laxity upon RTS after ACL‐R. In contrast, several recent studies did not find a clinically meaningful relationship between the magnitude of knee hyperextension and postoperative knee laxity, subjective knee function or revision surgery risk [8, 28].

Despite inconsistent results reported among the most recent studies, there is a growing awareness of both GJH and knee hyperextension as potential risk factors for both primary ACL injury and reinjury risk after ACL‐R [128]. While knee hyperextension can be a component of GJH, some patients with GJH present without concurrent knee hyperextension, and some patients with knee hyperextension may not fulfil other criteria for the diagnosis of GJH. Therefore further clarification regarding the independent roles of hyperextension and generalized joint laxity on ACL revision risk is warranted. Current studies often fail to distinguish patients with joint hypermobility and patients with knee hyperextension and may confound the impact of each independent variable on ACL reinjury risk. In studies investigating both knee extension and GJH, the presence of knee hyperextensibility was reported to increase the predictive potential of the Beighton score for residual high‐grade pivot shift after ACL‐R [3]. Knee hyperextension ≥5° was reported to be present in one third of patients undergoing revision ACL‐R [38], which further highlights the need to clarify the independent role of knee hyperextension on the risk of ACL reinjury and inferior patient outcomes after ACL‐R.

## FEMORAL TORSION

**Figure 1:**
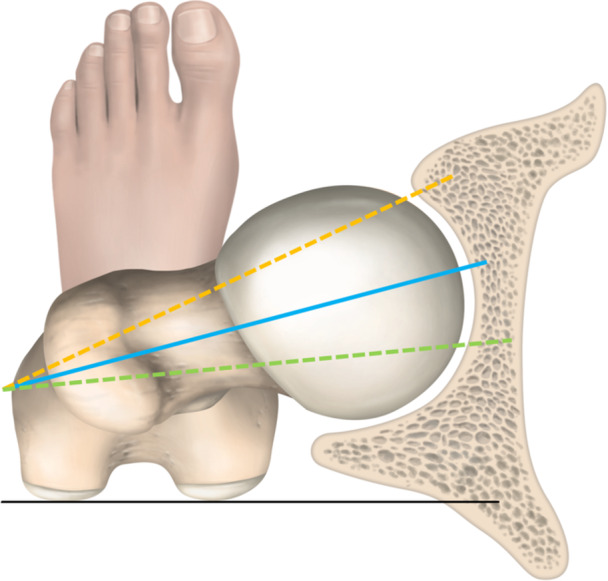
Schematic illustration of femoral torsion. The magnitude of femoral torsion is determined based on the angle enclosed by the distal and proximal axes of the femur, using various computer tomography‐based methods. While the axis of the distal femur is determined based on a tangential line (black line) against the posterior aspects of the femoral condyles across all methods, determination of the proximal axis varies based on the position of the transverse and oblique slices along the femoral neck. The figure illustrates femoral torsion measurements corresponding to the normal range (blue line), anteversion (orange dashed line) retroversion (green dashed line). Approximately 8°–15° of anteversion is reported to be physiologic in the adult population.

Femoral torsion refers to the three‐dimensional rotational alignment between the proximal and distal ends of the femur and is quantified by the angle measured between the two points in the transverse plane (Figure 1) [59]. Femoral neck anteversion (FNA) is characterized by the increase in the angle of the version between the proximal and distal axes of the femur. It can be reliably assessed based on several configurations of radiographic landmarks using computerized tomography (CT) and magnetic resonance imaging (MRI) [59, 111]. While the magnitude of femoral version is between 35° and 45° at birth, there is a successive decrease in angular torsion during the growth period [29, 129], with 8°–15° of anteversion considered physiologic in adults [29, 111]. Excessive anteversion of the femoral neck leads to reduced congruity of the femoral head and the hip joint. In turn, compensatory internal rotation to maintain hip joint congruity leads to functional valgus alignment of the knee joint, with detrimental effects on knee kinematics [60]. Biomechanical changes in patients with increased FNA consequently include alterations in the line of action, lever arms and activation of the hip abductors, with potential impact on hip and knee joint kinematics during movement patterns associated with non‐contact ACL injury [22, 73]. As a recent systematic review and meta‐analysis did not determine a conclusive effect of femoral anteversion and passive hip range of motion on ACL injury risk [51], further research is necessary to clarify the biomechanical impact of the anatomic characteristics of the femur on ACL reinjury risk specific to both male and female patients. Despite contrasting evidence, the functional and radiographic assessment of FNA may be motivated as part of the patient assessment for anatomical and biomechanical risk factors of ACL revision.

## INTERCONDYLAR NOTCH MORPHOLOGY

The intercondylar notch serves as a passageway for the ACL. Awareness of the influence of variation in intercondylar notch size and morphology, as well as the influence of this variation on the risk of ACL injury ACL‐R failure, may have important implications on revision ACL‐R risk stratification. Methods for the routine assessment of intercondylar notch morphology include measurements based on the preoperative MRI or measurements taken intraoperatively and are described in detail elsewhere [136].

**Figure 2:**
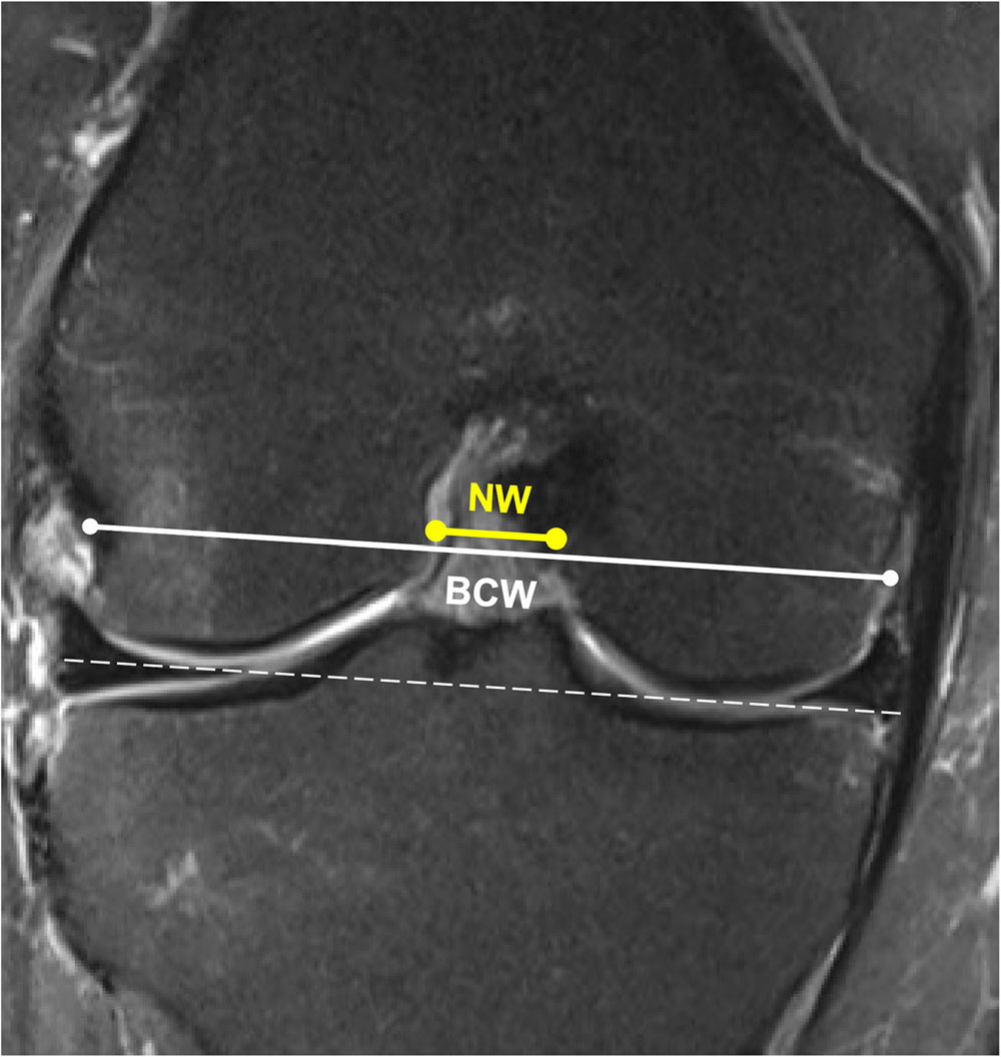
Measurement of the intercondylar notch width (NW) and intercondylar NW index (NWI) on a coronal magnetic resonance image. A line is drawn tangential to the distal aspects of the femoral condyles (dashed white line). The bicondylar width (BCW) is then determined through the superimposition of a parallel line at the intersection of the apex of the popliteus sulcus (white line). The NW (yellow line) corresponds to the width of the intercondylar notch at the level of the BCW. The NWI can subsequently be calculated by dividing the NW with the BCW.

A narrow intercondylar notch width (NW) has been reported as a significant risk factor for ACL injury (Figure 2) [4, 78, 107]. One meta‐analysis found that patients with ACL tears had significantly narrower NW compared with controls, consistently observed across different ethnic groups and patient sex [78]. Furthermore, patients with ACL injury had smaller NWs than age‐ and sex‐matched patients without ACL injury [107]. Intercondylar NW < 16 mm (17.6% failure rate) was associated with a fivefold increase in the risk of ACL‐R graft failure compared with NW ≥ 16 mm (2.3% failure rate) at a short‐ to mid‐term follow‐up of patients with ACL‐R [52]. Variation among intercondylar notch phenotypes may further help stratify patients at increased risk for ACL‐R graft failure [4, 9, 153]. An A‐type notch, characterized by a stenotic profile that narrows from the midsection to the base and apex, has been associated with an approximately 3‐fold increase in ACL injury risk compared with the wider type U and W notches [9]. Based on a more complex three‐dimensional morphological classification, a notch profile with both inlet and outlet stenosis was associated with smaller notch volume and greater ACL injury risk [153]. The NW index (NWI), defined as the ratio of the intercondylar NW and the femoral bicondylar width, is an additional metric used to characterize intercondylar notch morphology [4, 16, 54, 78]. While some studies report a smaller NWI to be associated with increased ACL injury risk [54, 113], others found no relationship between NWI and the prevalence of ACL injury [16]. Interestingly, one matched‐control study [107] found an increasing NW, NWI, and medial tibial depth with respect to age in the ACL‐injured cohort, irrespective of patient sex. These findings may suggest a dynamically changing impact of bone morphology on ACL injury risk over the course of bone development, which may partially explain the role of patient age as a covariate in revision ACL‐R risk assessment. Despite the inconclusive evidence, there is a rationale for the potential contribution of intercondylar notch morphology to ACL injury risk based on the discussed anatomic parameters [4].

While a combined radiological assessment of bone morphologic parameters is proposed to result in more robust risk prediction for ACL injury, more than 50% of healthy individuals are estimated to present with a tibial or femoral risk factor for ACL injury [42, 89]. Consequently, large cohort studies are required need to assess the validity of tibiofemoral bone morphologic risk factors for ACL‐R failure risk prediction [88]. Despite the described biomechanical implications of intercondylar notch morphology, clinical studies of primary ACL‐R where concurrent notchplasty was performed have not shown favourable patient outcomes or reduced graft rupture rates compared with ACL‐R alone [41].

In summary, specific intercondylar notch characteristics may create unfavourable biomechanical conditions for the native ACL and the ACL graft, which may in turn predispose patients to primary ACL injury and graft failure following ACL‐R. Knee surgeons are advised to consider ACL graft diameter in relation to intercondylar notch morphology and dimensions to assess the potential risk of revision ACL‐R due to graft impingement [34, 136]. Future research utilizing standardized, three‐dimensional assessment methods of notch morphology and longitudinal clinical data may help clarify the relationship between intercondylar notch characteristics and revision ACL‐R risk.

## LATERAL FEMORAL CONDYLE (LFC) MORPHOLOGY

LFC morphology has received increasing attention over recent years with regard to its impact on ACL injury risk. Several studies discuss the role of the LFC ratio (LFCR), defined as the ratio of the posterior femoral condylar depth to total condylar length, on the risk of ACL injury and graft failure. An increased (>63%) LFCR measured on lateral radiographs has been associated with primary ACL injury, failed ACLR, and contralateral ACL injury [101]. Similar results were reported based on MRI measurements, with a positive association between increased LFCR and ACL injury and ACL reinjury [43, 124]. An increased LFCR is hypothesized to be a contributing factor to abnormal tibiofemoral interactions, altered gait and loading mechanics [101], which are associated with increased ACL injury risk [45, 46]. An increased LFCR may also contribute to the increased tautness of the anterolateral structures of the knee in flexion and therefore greater laxity close to full extension [101]. Consequently, the ability of the anterolateral structures to maintain rotatory stability near full knee extension (a position where the ACL is particularly vulnerable to injury) may be compromised [75, 101]. Furthermore, an increased LFCR is associated with a greater risk of anterolateral complex injury in patients with non‐contact ACL injury [75], with a potential exacerbation of rotatory knee laxity and a further increase in the risk of ACL injury [102]. Consequently, a positive association between LFCR and increased rotatory laxity in patients with ACL injury may warrant future assessment of the effect of extraarticular soft‐tissue procedures to reduce reinjury risk in patients predisposed to rotatory instability [75, 101].

The distal curvature of the LFC is an additional morphologic factor with a potential impact on ACL injury and reinjury risk. A decreased ratio of LFC height to anteroposterior diameter has been associated with a greater risk of ACL injury [76]. Similarly, an elongated or flattened LFC has been associated with increased ACL injury risk [32]. Additionally, associations between posterior femoral depth and an increased magnitude of rotatory knee laxity [102], as well as increased LFC depth and multiple ACL‐R failures, were reported [36]. While further research is required to verify the role of bone morphologic parameters with respect to revision ACL‐R risk, awareness of LFC morphology as a potential anatomic risk factor for ACL‐R failure may help surgeons guide initial management and identify patients at risk of reinjury due to anatomic variation.

## POSTERIOR TIBIAL SLOPE (PTS)

**Figure 3:**
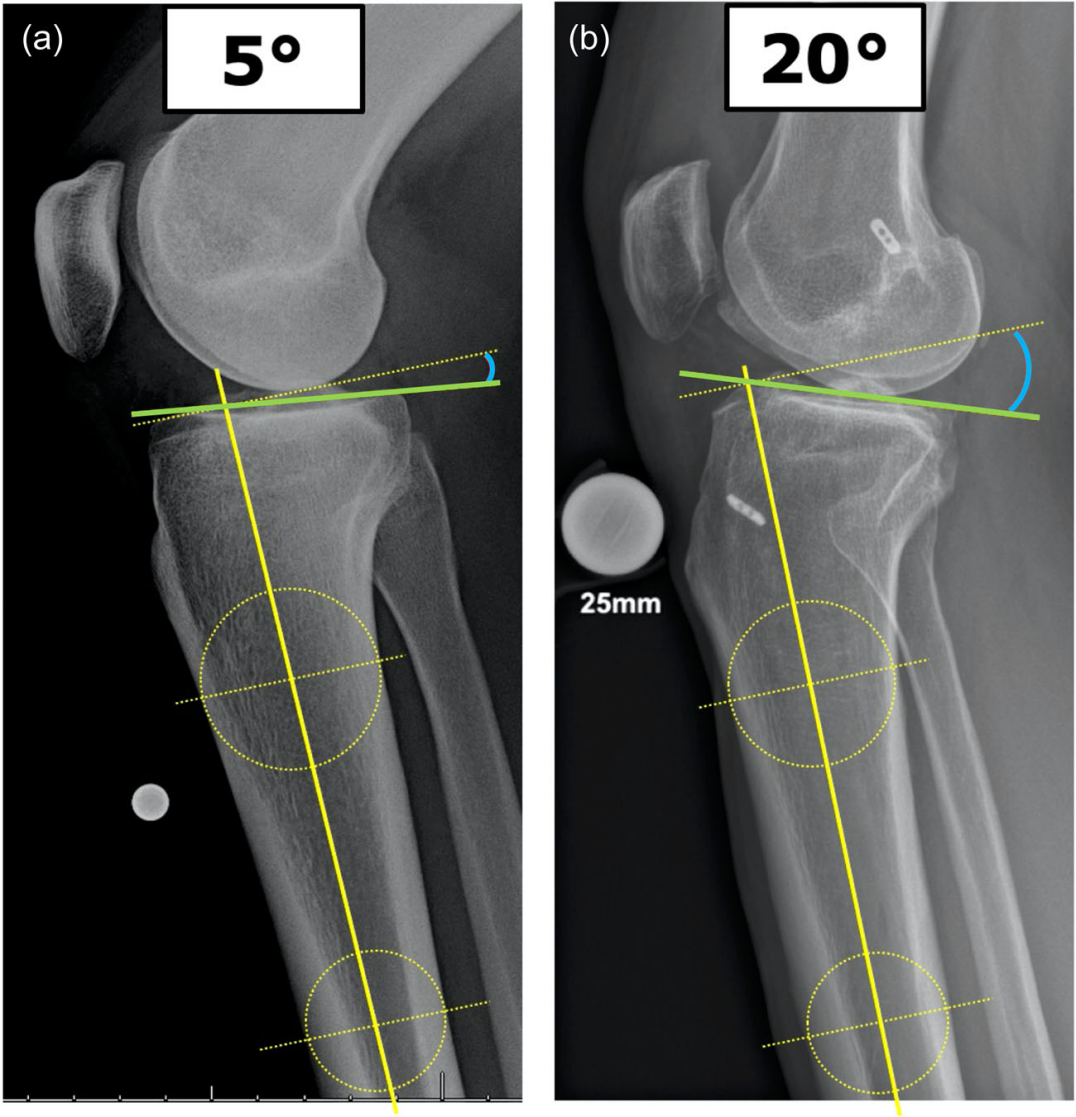
The posterior tibial slope (PTS) can be determined based on lateral knee radiographs. Two concentric circles (yellow dotted circles) are superimposed on the tibia approximately 5 and 10 cm distal to the tibiofemoral joint line. A vertical line transecting the centres of the two circles is drawn to determine the anatomic axis of the tibia (vertical yellow line). The angle (blue line) between a line drawn perpendicular to the anatomic tibial axis (yellow dotted line) and another drawn tangential to the tibial plateau (green line) corresponds to the magnitude of the PTS. (a) A PTS of 5° is considered normal and does not increase the risk of primary ACL injury or ACL revision. (b) A PTS of 20° may lead to unfavourable knee kinematics and greater anteroposterior tibial translation, which leads to an increased risk of both primary ACL injury and ACL revision. ACL, anterior cruciate ligament.

Over the past decade, the contribution of the PTS (Figure 3) to primary ACL injury and revision ACL‐R risk has increasingly been gaining attention [138]. Based on several biomechanical and clinical studies, PTS has been recognized as a modifiable risk factor for both primary and recurrent ACL injury [18, 25, 138]. The fundamental concept behind the role of the PTS on ACL injury risk is the assumption that a greater magnitude of PTS results in increased tibial shear force, followed by increased anterior tibial translation, and increased in‐situ forces in the ACL [53, 112, 142]. An osteologic study of PTS magnitude in 1090 tibiae found a range of PTS between −8.4° and 18.7° [146]. Given the broad variation in the magnitudes of the medial and lateral PTS between men and women, as well as ethnic groups, the PTS has been dubbed the ‘fingerprint of the tibial bone’ [149]. A recent study compared the PTS of 1000 ACL‐intact and 1000 ACL‐injured knees, with a significantly higher mean PTS reported in the patient population with ACL injury (10.0 ± 3.0° vs. 9.0 ± 2.9°) [145]. While the PTS difference across the entire patient cohort may not appear to be clinically relevant, it was also shown that a significantly higher proportion of patients with ACL injury had a PTS of 12°or greater (32% vs. 20%), compared with patients without ACL injury [145]. Accordingly, a PTS of 12–14° measured on lateral knee radiographs is often considered the threshold above which there is an increased risk of primary ACL injury, first‐time ACL‐R failure and repeat ACL‐R failure [25, 145]. Moreover, increased PTS may be a risk factor for medial and lateral meniscal body and meniscal root injuries, potentially increasing the risk of ACL‐R failure [26, 57]. Accordingly, PTS‐modifying osteotomies have become a part of the repertoire of ACL surgeons and show promising short‐term clinical results with a low failure rate of around 2% based on recent reports [62, 91, 150]. Despite the encouraging results of combined ACL‐R and PTS‐modifying osteotomy, the invasiveness of the additional osteotomy, the extended operative time, the effect of PTS modification on the patellofemoral joint, and the reported complication rate of up to 57% have to be recognized [62, 91]. One recent study of patients with isolated single‐bundle ACL‐R without concomitant injuries (*N* = 326) found no PTS difference between patients with (10.6 ± 3.2°) and without (11.2 ± 2.8°) ACL graft failure and no association between PTS and patient‐reported outcome scores [49]. In paediatric patients (≤18 years of age), a recent meta‐analysis found no association between PTS and ACL injuries [30]. Consequently, the role of PTS remains a controversial topic that requires further assessment to establish a direct impact on revision ACL‐R risk.

## TIBIAL MORPHOLOGY

While the PTS is well‐studied in terms of both ACL injury and ACL‐R failure risk, additional morphologic characteristics of the tibia are less established risk factors, despite their potential influence on postoperative outcomes and ACL‐R failure. Several aspects of tibial morphology, including the dimensions and shape of the tibial plateau, the intercondylar spine, and the associated soft tissue structures such as the cartilage and meniscus, are reported to affect both primary ACL injury risk and the risk of ACL‐R failure [4, 17, 121, 131].

A smaller anteroposterior and mediolateral tibial plateau diameter, which overall results in a smaller total tibial plateau size, was reported as a risk factor for ACL injury [4, 92, 103]. A reduced tibial plateau size may impact the biomechanical stability of the knee joint, through a potential increase in ACL graft stress following ACL‐R. In addition to a smaller lateral tibial plateau size, increased tibial plateau convexity is also reported in association with increased objective pivot shift and greater ACL injury risk [70]. One study found that patients with ACL injury have a smaller tibial plateau radius of curvature and greater articular surface convexity compared with activity‐matched athletes without ACL injury, which implies reduced knee stability during anterior tibial translation and tibial rotation [139].

Furthermore, the shape of the intercondylar tibial eminence also seems to be associated with ACL injury risk. Several studies report that a smaller tibial eminence width is associated with an increased ACL injury risk [4, 134, 151]. The interaction between bone and soft tissue structures like the meniscus and cartilage also seems to play an important role in the context of tibial plateau morphology [122, 131, 135]. Measurements of tibial plateau morphology, menisci and articular cartilage revealed a complex interaction in terms of ACL injury risk [121, 122]. A steeper meniscus‐to‐bone angle and meniscus‐to‐cartilage angle, defined as the angle between the anterior face of the posterior meniscus horn and the tibial cartilage or bone, as well as an increased meniscus‐to‐cartilage height, seem to be protective against ACL injury [122, 131, 135]. The explanation for the protective role of the described factors is the opposing force to anterior translation exerted by the meniscus (as a function of height and angle) through its function as a wheel chock against the convex femoral condyles.

**Figure 4:**
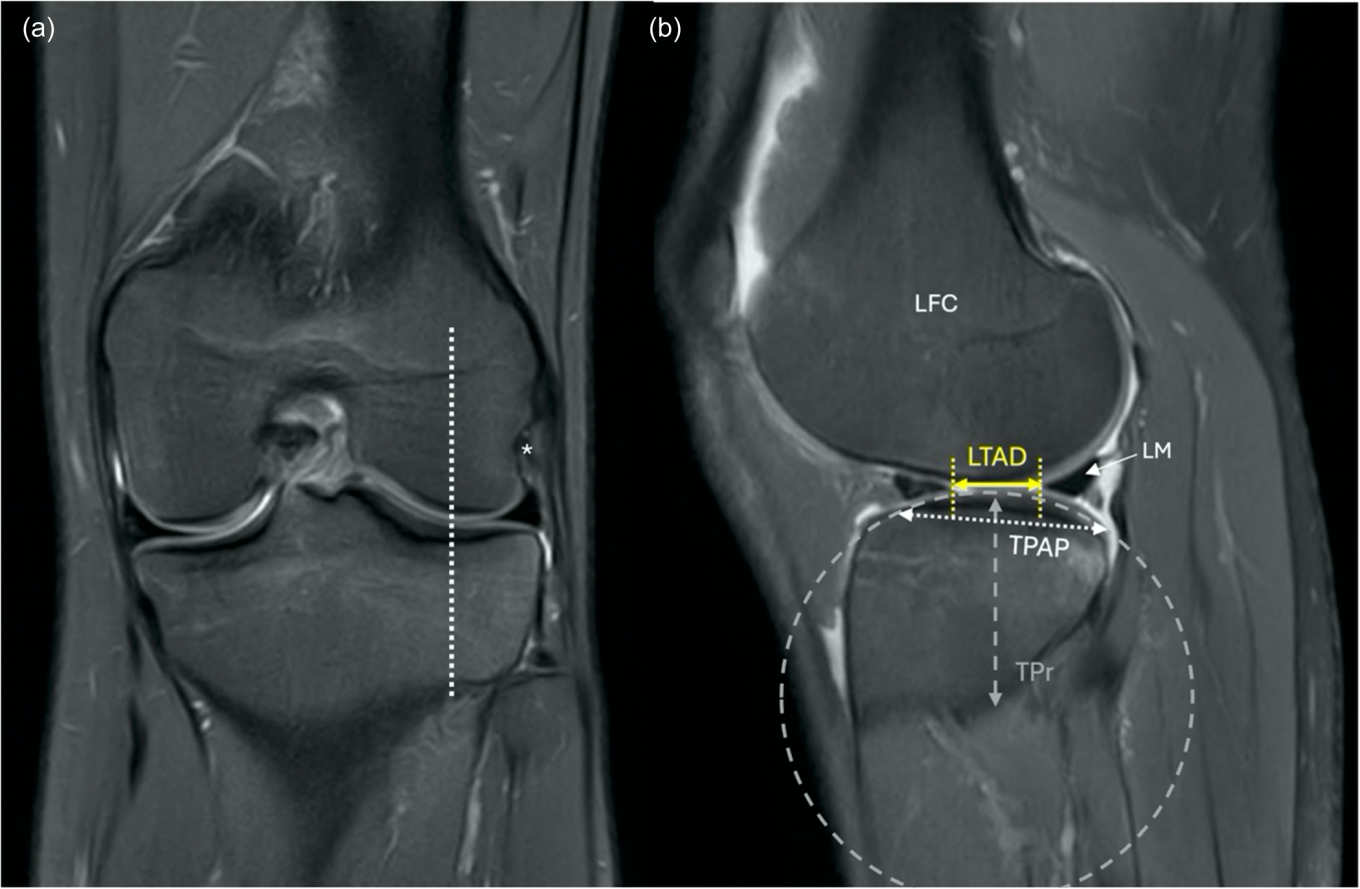
(a) The midsagittal plane of the lateral femoral condyle (LFC) is located at the level of the popliteal femoral insertion point (*) on a coronal magnetic resonance image. Identification of the midsagittal plane of the femur involves locating the vertical axis (represented by the dashed white line) that intersects the most inferior point of the convex surface of the articular cartilage at the distal femur. This axis generally aligns close to the midpoint of the mediolateral articular width on the lateral side of the joint. (b) The anteroposterior distance between the most posterior point of the anterior horn and the most anterior point of the posterior horn of the lateral meniscus (LM) is referred to as the lateral tibiofemoral articular distance (LTAD). The tibial plateau anteroposterior length (TPAP) represents the length of the articular surface measured along the subchondral plate. To characterize the curvature of the proximal tibial articular surface, a best‐fit circle (grey) is superimposed. The tibial plateau radius of curvature (TPr) is defined as the radius of this circle. A smaller LTAD, a shorter TPAP and a smaller and more convex TPr are potential risk factors for ACL injury. ACL, anterior cruciate ligament.

Recently introduced, the lateral tibiofemoral articular distance (LTAD) may be used as a simple proxy measurement of tibiofemoral contact area in the lateral knee compartment (Figure 4), which accounts for both bony and soft tissue factors with a contribution to knee stability [17]. The LTAD is a measure of both the convexity and morphology of the femoral condyle, tibial plateau, as well as the meniscal volume in the lateral compartment, with a strong correlation to tibial acceleration during the pivot shift manoeuvre [17].

While the aforementioned tibial morphological risk factors for ACL injury and graft rupture are well‐documented in the literature, exact thresholds for the stratification of individuals at risk of re‐injury, as well as the specificity and sensitivity of the various parameters, remain to be determined. One recent study demonstrated that following the application of thresholds commonly reported in the literature to a healthy reference population, 15%–62% of individuals were classified at risk of ACL injury [88]. Consequently, this method may lack precision and could lead to an overestimation of ACL injury risk based solely on the currently reported values for anatomic parameters.

While the majority of the discussed morphological factors are either non‐modifiable or only minimally modifiable, recognition of their impact on ACL‐R failure risk may help surgeons plan management and whether additional surgical procedures, such as extra‐articular tenodesis, are warranted. Overall, the influence of tibial bony morphology, as well as the soft tissue properties on tibiofemoral congruence and stability, require further clarification. In particular, quantitative assessment of the complex interaction between the individual morphologic parameters may help stratify patients with an increased risk for ACL‐R failure.

## GENETIC FACTORS

Familial studies in the population with ACL injury have raised awareness of the hereditary component of ACL injury. Individuals with first‐degree relatives with an ACL injury are exposed to a 1.79‐fold risk of ACL injury [1]. Other studies consistently report the increased risk of ACL injury among the relatives of patients with both unilateral [33] and bilateral [40] ACL injuries. Interestingly, a study of 88,414 identical and fraternal twins from the Swedish Twin Register determined an approximately 69% heritability of ACL injury [84], which implies a strong genetic contribution to ACL injury risk. A case series of four brothers with consecutive ACL injuries before the age of 22 years further corroborates the causal role of heritable factors based solely on probability, as the chance of occurrence of this event is estimated to be 1 in 36 million [61]. While the previous examples are strongly suggestive of the impact of genetics on ACL injury risk, recent research aims to clarify the role of specific genetic variants on ACL injury risk at the molecular level, with a focus on genes involved in the regulation of the structural characteristics of ligaments, inflammatory responses, extracellular matrix remodelling, and tissue repair mechanisms (Table 2).

**Table 2:** A non‐exhaustive list of gene polymorphisms potentially associated with ACL injury risk.

Study	Gene(s)	Polymorphism (SNP ID)	Genotype/allele	Association with ACL injury	OR	95% CI	Population studied
Collagen genes							
Guo et al. [39]	*COL1A1*	*rs1800012*	TG vs. GG	Increased risk	1.25	1.02–1.55	N/A
Guo et al. [39]	*COL1A1*	*rs1800012*	TT vs. GG	Protective	0.53	0.34–0.83	Caucasian
Guo et al. [39]	*COL1A1*	*rs1800012*	TT vs. TG + GG	Protective	0.50	0.32–0.78	Caucasian
Posthumus et al. [106]	*COL5A1*	BstUI RFLP	CC genotype (in female patients)	Protective (underrepresented in female patients with ACL injury)	6.6	1.5–29.7	Caucasian
Posthumous et al.[105]	*COL12A1*	AluI RFLP	AA genotype (in female patients)	Increased risk (over‐represented in patients with ACL injury)	2.4	1.0–5.5	Caucasian
Inflammatory response genes							
Lorenz et al. [81]	*IL6*	*rs1800795*	GC genotype	Increased Risk	1.30	1.02–1.66	N/A
Matrix metalloproteinases genes							
Šimunić‐Briski et al. [116]	*MMP3*	*rs591058*	TT genotype	Increased Risk	3.86^a^	1.70–8.73^a^	Croatian athletes
Šimunić‐Briski et al. [116]	*MMP3*	*rs650108*	GG genotype	Increased Risk	2.33^a^	1.29–4.22^a^	Croatian athletes
Šimunić‐Briski et al. [116]	*MMP3*	*rs679620*	AA genotype	Increased Risk	3.48^a^	1.53–7.91^a^	Croatian athletes
Vascular endothelial growth factor A							
Feldmann et al. [31]	*VEGFA*	*rs2010963*	CC genotype	Increased Risk	2.16	1.47–3.19	Multiple populations
Li et al. [77]	*VEGFA*	*rs699947*	AA and AC genotypes	Protective^a^	0.92	0.86–0.98	European
Li et al. [77]	*VEGFA*	*rs1570360*	AA and GG genotypes	Increased risk^a^	1.29	1.14–1.45	European
Li et al. [77]	*VEGFA*	*rs1570360*	G allele	Protective^a^	1.15	1.00–1.32	European
Li et al. [77]	*VEGFA*	*rs1570360*	GG genotype	Increased risk^a^	1.40	1.00–1.94	European
Other							
Dlamini et al. [24]	*ITGB2*	*rs2230528*	CC vs. TT	Overrepresented in control group without ACL‐R	N/A	N/A	In silico
Dlamini et al. [24]	*ITGB2*	*rs2230528*	TT vs. CC	Overrepresented in the ACL‐R group	N/A	N/A	In silico

Abbreviations: ACL, anterior cruciate ligament; ACL‐R, anterior cruciate ligament reconstruction; CI, confidence Interval; N/A, not applicable; OR, odds ratio; RFLP, restriction fragment length polymorphism; SNP ID, single‐nucleotide polymorphism identifier; VEGFA, vascular endothelial growth factor A.

a Tendon and ligament injury, not ACL exclusively.

Genes involved in collagen synthesis, particularly ones encoding types I and V collagen [5, 39, 109, 123], have been studied in the context of ACL injury risk due to their involvement in the synthesis of the structural components of ligaments. Several studies found specific single nucleotide polymorphisms in genes coding for the α1 chains of types I, V and XII collagen, specifically *COL1A1* [104], *COL5A1* [106] and *COL12A1* [105] genes, to be over‐ or underrepresented in subpopulation of patients with ACL injury, with respect to ethnic or sex‐based variation. Specific genetic variations may therefore be important to understanding the multifaceted role of allelic variation in the aetiology and risk of ACL injury and revision ACL‐R. One meta‐analysis determined that the TT genotype of the *rs1800012* polymorphism in the *COL1A1* is associated with a protective effect against musculoskeletal soft tissue injuries, including ACL injury [39]. However, evidence there is limited evidence to support the impact of collagen synthesis genes, as several studies report no clinically relevant associations between candidate polymorphisms of the *COL1A1*, *COL3A*, *COL5A1*, *COL12A1* genes and ACL injury risk [87, 117].

Genes involved in the regulation of the inflammatory cascade are also hypothesized to impact ACL injury risk [81, 83, 123]. Meta‐analysis of studies on the assessment of polymorphisms in genes encoding interleukins found the *rs1800795* CG genotype of the *IL6* gene was overrepresented in patients with ACL‐R (odds ratio [OR] = 1.30), while the *rs16944* CT genotype of the *IL1B* gene was associated with reduced risk of ACL‐R (OR = 0.89) [81]. Further studies are warranted to assess the potential effect of genes involved in the regulation of inflammatory cytokines and revision of ACL‐R risk.

Additionally, polymorphisms in matrix metalloproteinases (MMPs) genes may be linked with ACL injury risk [82, 116]. In a cohort of 187 Croatian athletes with and without ACL injury, three distinct *MMP3* polymorphisms were associated with increased odds of noncontact ACL injury [116]. Conversely, another study did not find associations between *MMP1*, *MMP10* and *MMP12* polymorphisms and noncontact ACL injury risk [82], which suggests further studies are required to determine the direct impact of variants in matrix metalloprotease genes and ACL injury and revision ACL‐R risk.

Vascular endothelial growth factor A (VEGFA) plays a critical role in angiogenesis and tissue repair. Several studies report associations between *VEGFA* gene polymorphisms and ACL injury risk [31, 77, 108, 114]. While meta‐analysis of studies assessing the impact of *VEGFA* polymorphisms on tendon and ligament injury risk found no direct genetic associations, subgroup analysis of the European population revealed three genotypes to be associated with a greater risk of tendon and ligament injury [77]. Specific *VEGFA* genotypes were over‐ and underrepresented in patients with ACL injury compared with healthy controls, which suggests certain single‐nucleotide polymorphisms to be directly associated with detrimental and protective effects on ACL injury risk, respectively [31]. Consequently, the potential influence of *VEGFA* gene polymorphisms on revision ACL‐R risk through the modulation of ligament and graft biology through angiogenesis may require further attention to stratify patients with increased susceptibility to graft failure.

Genome‐wide association studies (GWAS) determined associations between genetic variants in high‐level athletes and musculoskeletal injuries [27]. One GWAS identified variants in the *ITGB2* and *FGF9* genes to be associated with susceptibility to ACL injury [24], and another failed to replicate previously observed associations between polymorphisms of genes involved in collagen, MMP and VEGFA synthesis and ACL injury [13]. As a result, inconsistencies in the proposed roles of genetic variants on primary ACL injury risk need to be resolved prior to further research on their potential impact on revision ACL‐R risk.

## MUSCLE STRENGTH IMBALANCE

**Figure 5:**
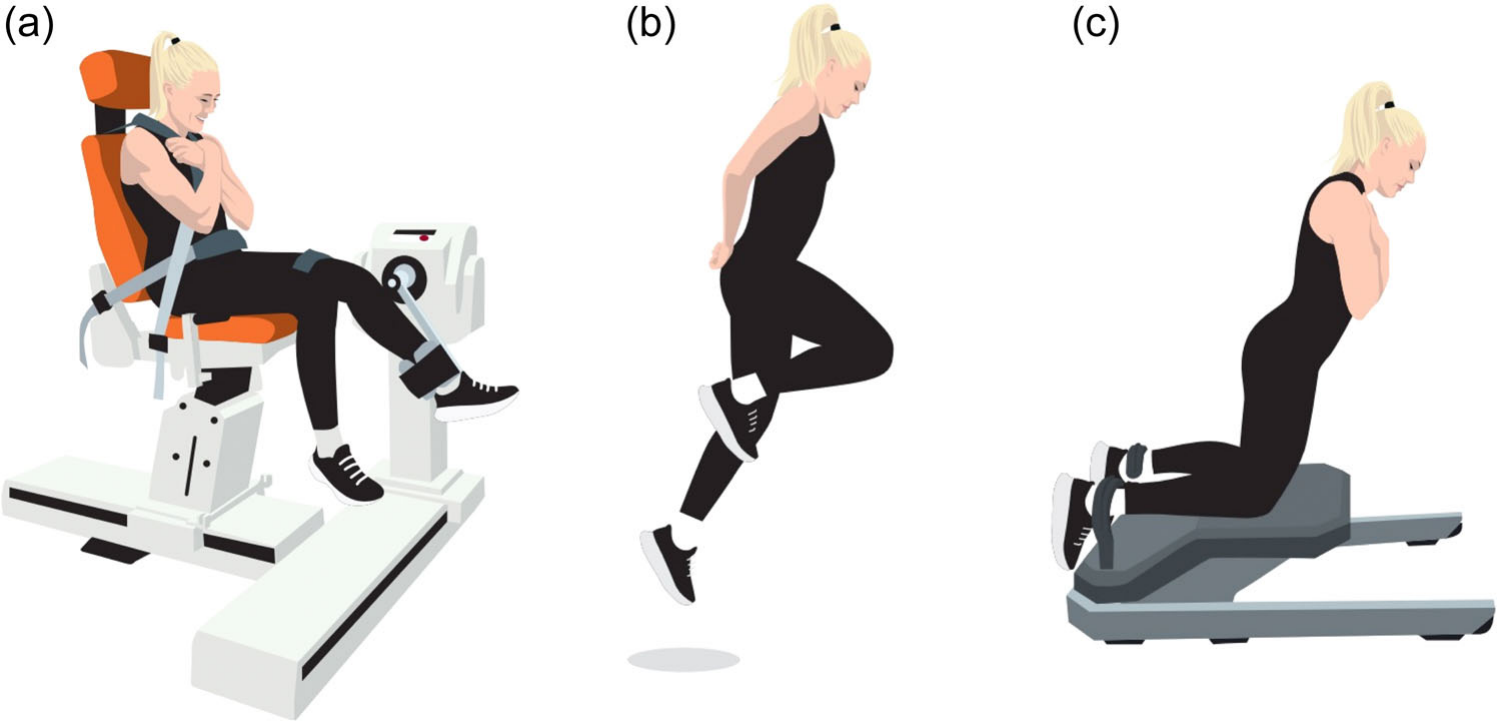
Schematic illustration of muscle function tests in patients with ACL injury. (a) Isokinetic dynamometer for the measurement of concentric extension and flexion strength; (b) single‐leg hop test; (c) eccentric hamstring strength test. ACL, anterior cruciate ligament.

Muscle strength imbalance between knee extensors and flexors may be a critical factor for ACL injury and subsequent revision ACL‐R risk. Quadriceps contraction induces anterior tibial translation, which increases strain on the ACL [120]. In contrast, the hamstrings counteract anterior tibial translation through co‐contraction [98]. The balance in the synergistic functional relationship between the quadriceps and hamstring muscles is characterized by the hamstring‐to‐quadriceps (H:Q) strength ratio and is regarded as an important contributor to knee stability (Figure 5). This is particularly true for high‐stress activities like jumping and cutting, which are common in many sports associated with an increased non‐contact ACL injury risk.

Accordingly, persistent H:Q muscular imbalance following ACL‐R may be considered a risk factor for revision ACL‐R. Current studies highlight the impact of greater quadriceps strength in relation to hamstring strength as a risk factor for ACL reinjury, presumably due to altered knee kinematics and increased strain on the ACL graft due to excessive anterior tibial translation [6, 96]. Patients with ACL‐R have inferior mean H:Q strength ratio compared with healthy controls, where 16% of the variance in H:Q strength ratio could statistically be attributed to ACL‐R [64]. In a recent study of 145 female football players, new‐onset noncontact ACL injury was associated with a lower H:Q ratio and greater knee extension strength [130].

However, current evidence is conflicting with regards to the impact of H:Q muscle strength imbalance, as another study of 574 patients showed that the H:Q strength ratio is not associated with increased odds of ACL reinjury [50]. Asymmetrical loading patterns reported in athletes who RTS after ACL‐R may be attributed to muscle imbalances, including quadriceps dominance or insufficiency, and are associated with ACL reinjury risk [100]. Additionally, the neuromuscular deficit was reported in athletes who fail to meet RTS criteria after ACL‐R and is also associated with increased ACL reinjury risk [23]. Furthermore, a reduced magnitude of hamstring peak torque was reported in patients with graft failure after ACL‐R, while no difference was reported in quadriceps peak torque in the same population [71]. Consistently, a 10% decrease in the H:Q muscle strength corresponded to a 10.6‐fold increase in the risk of ACL graft rupture after RTS [71].

The quadriceps muscle group is critical to dynamic joint stability, and weakness and asymmetry of the muscle group compared with the contralateral knee are associated with poor functional outcomes after ACL‐R [15, 37, 63, 67, 115]. Younger athletes who participate in high‐demand sports often develop considerable quadriceps dominance due to activity‐specific demands [55]. Sports that emphasize explosive power and speed often lead to stronger quadriceps compared with hamstring musculature, which can potentially compromise knee stability and increase the risk of ACL injury and revision ACL‐R [47]. Despite adequate rehabilitation, the rate of force development (RFD) of the quadriceps muscles may be impaired in patients with RTS after ACL‐R [133], with a negative impact on athletic performance and reinjury risk [12]. Increased reaction time due to delayed quadriceps peak force development may consequently impair agility and increase the likelihood of suboptimal movement patterns that lead to ACL reinjury [99]. While current evidence regarding the impact of persistent muscle strength imbalance of ACL reinjury risk is conflicting [50, 115], targeted intervention to correct relative hamstring and quadriceps strength may to some extent contribute to injury risk reduction in patients with functional knee instability after ACL‐R.

## DYNAMIC KNEE VALGUS AND HIP STRENGTH

Valgus alignment of the knee is characterized by the lateral angulation of the tibia relative to the femur in the frontal plane. The described angular deformity leads to increased load on the lateral compartment of the knee joint, with an important impact on knee kinematics. Knee valgus may be present under static or dynamic conditions. The latter involves cutting, landing, or pivoting activities, which are prevalent injury patterns associated with ACL injury [20]. Additional factors associated with knee valgus during athletic activity include, but are not limited to trunk positioning and hip strength [148]. Lateral trunk lean and hip internal rotation are significantly correlated with increased knee valgus during landing tasks [137]. Hip abductor and hip external rotator weakness have a detrimental effect on control of the femur, contributing to the inward collapse of the knee during dynamic knee valgus. Furthermore, decreased hip abduction strength is associated with knee valgus and may contribute to the incidence of primary ACL injury [65]. However, the potential contribution of quadriceps weakness, decreased eccentric quadriceps function, slow quadriceps RFD, or a combination of the above to knee valgus loading should also be considered. An athlete with poor quadriceps function may struggle with rapid deceleration (rapid eccentric flexion) and may be predisposed to valgus collapse of the knee and subsequent ACL injury [56]. Dynamic knee valgus is prevalent in young females and is likely an independent contributor to the high rates of ACL injury in this patient population [46, 68]. Consequently, interventions targeting strength and neuromuscular control at the hip may potentially decrease primary ACL injury and revision ACL‐R risk [95].

Patients with ACL‐R often fail to achieve optimal recovery of abduction and adduction strength at the 6‐8‐month postoperative follow‐up [11]. Early RTS without adequate recovery of hip muscle strength and persistent dynamic knee valgus may therefore expose athletes to a greater risk of ACL reinjury and subsequent revision ACL‐R [100]. However, hip strength assessment is rarely a component of RTS assessment after ACL‐R at present [48]. Rehabilitation protocols may therefore benefit from the consideration of the persistent imbalances that may contribute to dynamic knee valgus after ACL‐R, to delay RTS and potentially reduce the risk of revision ACL‐R.

## ACTIVITY LEVEL AND INJURY RISK EXPOSURE

Activity level is considered an important risk factor for primary ACL injury and likely affects reinjury risk exposure after ACL‐R. High‐intensity sports with frequent cutting, pivoting, and jumping movements, such as football, basketball, American football and skiing exert substantial stress on the knee and the ACL [90]. Sports that require sudden changes in direction and rapid deceleration are particularly risky, especially since sudden changes of direction and deceleration require proper RFD. Following ACL‐R, the quadriceps rarely recover proper RFD [12]. The resultant forces exceed the tensile strength of the ACL under conditions when there is inadequate restraint provided by the muscle groups about the knee joint, and result in ligament injury. For instance, the incidence of ACL injuries in gymnastics [90] or football players [141] is notably high due to the constant need for rapid directional changes and high‐speed movements, subjecting the quadriceps to high demands. Furthermore, athletes with greater training volumes and intensities are more likely to suffer ACL injuries [2]. Overuse and fatigue can impair neuromuscular control, making athletes more susceptible to injury during high‐demand movements [10]. Thus, athletes with high activity levels and with increased activity‐associated exposure are likely to be more prone to ACL injury.

The current literature highlights that athletes who returned to their pre‐injury level of sports activity are exposed to a greater risk of re‐injury [143]. Strenuous sport‐specific demands over time, rather than the isolated event of RTS, are likely the causal factors behind ACL re‐injury risk [143]. Accordingly, high‐demand sports are associated with an increased risk of ACL graft rupture or injury to the contralateral ACL [143]. The synergistic effect of time and exposure type is therefore essential to understand and assess reinjury risk in patients with ACL‐R.

Both the duration and intensity of exposure may influence the risk of ACL reinjury. Athletes who engage in frequent high‐intensity training sessions or matches are at greater risk due to the cumulative stress exerted on the knee joint. This is particularly true in younger athletes with high volumes of match exposure due to their involvement in multiple competitions, tournaments or leagues [21, 147]. Additionally, match exposure likely corresponds to a greater risk compared with training exposure due to the unpredictable and competitive nature of matches, where rapid changes in direction, deceleration and high‐speed collisions are more frequent. Increased exposure time, particularly in matches, is directly associated with a greater overall injury incidence [35, 140]. Consequently, the management of activity frequency, intensity and duration, with consideration to both training and match exposure is crucial to mitigate ACL injury and revision ACL‐R risk. Previous research has shown that young athletes aim to RTS as early as possible after ACL‐R [144]. A strong sense of identity derived from athletic participation may further motivate young athletes to disregard known risk factors for injury and is associated with persistent risk exposure and reinjury risk following the index injury [74, 80].

## CONCLUSION

In conclusion, we strongly advocate that the synergistic effect of modifiable and non‐modifiable factors presented herein may lead to early ACL revision and the selective attrition of high‐risk patients included in registry cohorts assessed for predictive factors associated with ACL revision risk. In turn, patient age as an independent factor might not be representative of ACL revision risk to the extent suggested by the current literature. Greater attention should instead be turned towards anatomic variation in bone morphology, genetic and physiologic patient phenotypes, activity level, injury risk exposure and muscle function for a more comprehensive and individualized risk assessment of ACL revision risk. It is important to emphasize that the presented risk factors may be present in isolation or in combination in patients with ACL injury, and may considerably modify ACL revision risk with respect to the individual patient. Finally, while the growing magnitude of data in patient registries presents several opportunities to optimize ACL revision risk assessment, future initiatives should aim to improve the completeness and granularity of registered data, augmented with variables that inherently magnify the risk of ACL revision in patients with ACL‐R and thereby provide clinically relevant insights to guide patient management.

## AUTHOR CONTRIBUTIONS

All listed authors have contributed substantially to this work: review of the literature, and primary manuscript preparation were performed by Bálint Zsidai, Ramana Piussi, Philipp W. Winkler, Armin Runer, Pedro Diniz and Riccardo Cristiani. Editing and final manuscript preparation were performed by Bálint Zsidai, Eric Hamrin Senorski, Volker Musah, Michael T. Hirschmann, Romain Seil and Kristian Samuelsson. All authors have read the final manuscript and given final approval of the manuscript to be published. Each author consented to be accountable for all aspects of the research to ensure that questions related to the accuracy or integrity of any part of the work are appropriately investigated and resolved.

## CONFLICTS OF INTEREST STATEMENT

Volker Musah reports educational grants, consulting fees and speaking fees from Smith & Nephew plc, educational grants from Arthrex and DePuy/Synthes, is a board member of the International Society of Arthroscopy, Knee Surgery and Orthopaedic Sports Medicine (ISAKOS), and deputy editor‐in‐chief of Knee Surgery, Sports Traumatology, Arthroscopy (KSSTA). Michael T. Hirschmann is a consultant for Medacta, Symbios and Depuy Synthes and is the editor‐in‐chief of Knee Surgery, Sports Traumatology, and Arthroscopy (KSSTA). Kristian Samuelsson is a member of the board of directors for Getinge AB (publ). The remaining authors declare no conflicts of interest.

## ETHICS STATEMENT

The ethics statement is not available.

## Data Availability

Data sharing is not applicable to this article as no data sets were generated or analysed during the current study.
